# Pulmonary adenoid cystic carcinoma: molecular characteristics and literature review

**DOI:** 10.1186/s13000-023-01354-4

**Published:** 2023-05-17

**Authors:** Zhixin Chen, Jiapeng Jiang, Ying Fan, Hongyang Lu

**Affiliations:** 1 The Second Clinical Medical College, Zhejiang Chinese Medical University, Hangzhou, 310053 P.R. China; 2 Zhejiang Key Laboratory of Diagnosis & Treatment Technology on Thoracic Oncology (lung and esophagus), Zhejiang Cancer Hospital, Hangzhou Institute of Medicine (HIM), Chinese Academy of Sciences, Hangzhou, Zhejiang 310022 P.R. China; 3 Department of Thoracic Medical Oncology, Zhejiang Cancer Hospital, Hangzhou Institute of Medicine (HIM), Chinese Academy of Sciences, Hangzhou, Zhejiang 310022 P.R. China; 4 Department of Medical Oncology, Lishui Municipal Central Hospital, Lishui, Zhejiang 323000 China

**Keywords:** PACC, Immunohistochemistry, Molecular characteristics, Diagnosis, Treatment, Prognosis

## Abstract

**Background:**

Pulmonary adenoid cystic carcinoma (PACC) is an exceptionally rare salivary gland-type malignant neoplasm. Because of its clinical manifestations, imaging features are not different from other types of non-small cell lung cancer, which is a diagnostic challenge for most doctors.

**Conclusions:**

A review of the literature shows that high amounts of immunohistochemical (IHC) markers, such as CK7, CD117, P63, SMA, CK5/6, and S-100 are helpful for PACC diagnosis. Surgical resection is the main treatment of PACC, but treatment options for advanced PACC patients are limited and the research of molecular targeted drugs is ongoing in advanced cases not eligible for surgery. Currently, research on PACC targeted therapy mainly focuses on the exploration of v-myb avian myeloblastosis virus oncogene homolog (MYB) and its downstream target genes. In addition, median tumor mutation burden and PD-1/PD-L1 were lower in PACC, which may indicate poor efficacy of immunotherapy in PACC patients. This review focuses on the pathologic features, molecular characteristics, diagnosis, treatment and prognosis of PACC to establish a comprehensive understanding of PACC.

## Introduction

Pulmonary adenoid cystic carcinoma (PACC), previously known as “columnar tumor”, usually originates from submucosal glands of the trachea and bronchus. PACC is clinically rare and constitutes one of the major types of salivary gland carcinoma affecting the lung [1, 2]. As a subtype of salivary gland cancer, PACC needs to be distinguished from other salivary gland tumors such as basal cell adenoma, pleomorphic adenoma, pulmonary mucoepidermoid carcinoma and metastatic adenoid cystic carcinoma of salivary gland. In addition, it is difficult to distinguish early PACC from common respiratory diseases such as chronic obstructive pulmonary disease and asthma due to its atypical clinical manifestations [3]. Thus, the correct diagnosis of PACC requires a comprehensive combination of clinical manifestations, imaging examination, immunohistochemistry, histopathology and mutation analysis. In the treatment of PACC, radical surgical resection is currently the main treatment strategy [4]. According to the extent and infiltration of the lesion, different surgical modalities are selected, supplemented by radiotherapy or chemoradiotherapy, and the effect of chemotherapy alone is weak. With the development of molecular pathology, the research on PACC has been increasingly extensive, mainly focusing on molecular targets.

## Epidemiological and clinical characteristics

Epidemiologically, PACC is a relatively rare malignant tumor, accounting for 0.04-0.2% of all primary lung malignant tumors [5], mainly originating from the small salivary gland in the tracheobronchial tree. PACC can occur at any age and is more common in the 40–60 age group [6]. PACC tends to occur more frequent in female [7, 8] and previous studies have reported that there is no significant relationship between PACC and smoking [9, 10]. The clinical presentation of PACC patients largely depends on tumor location and distal obstruction, and cough with no obvious cause is the first symptom in most patients, and other symptoms, such as dyspnea, wheezing, obstructive pneumonia, hemoptysis, fever, fatigue, dysphagia, chills, chest pain and weight loss [11–13], are not unique. At the same time, some cases show no symptoms and are incidentally discovered during physical examination. Therefore, it is easy to misdiagnose, leading to delay in treatment. The histology and biological behavior of PACC are similar to those of malignancies occurring in the salivary glands of the head and neck [14]. Thus, PACC is considered a low-grade slow-growing malignancy with a high incidence of peripheral nerve invasion and often shows a tendency to undergo submucosal extension [15]. When the thyroid gland is invaded by the tumor, hypothyroidism may occur [16]. Compression of the esophagus may lead to dysphagia and invasion of the recurrent laryngeal nerve may lead to hoarseness [17]. Resio et al. [18] reported 424 PACC patients, the lymph node metastasis rate was 38%, which is related to distant metastasis and reduced survival [19]. Intrapulmonary metastases are the most common metastatic sites, followed by bone, liver, and brain; rare metastasis sites are adrenal glands, thyroid glands, etc. [20].

## Imaging examination

PACC is a salivary gland tumor originating from the submucosal glands of the trachea and bronchus, and airway glands are mainly distributed in the trachea and the large bronchus; therefore, this disease is more common in the central type [21]. As with other tracheal neoplasms, PACC is often undetectable on conventional chest radiographs due to the overlap of mediastinal and skeletal shadows in the trachea. This problem can be avoided by applying computed tomography (CT). Li et al. [21] reported the CT results in 30 PACC patients, including 24 with the central type. Among the 24 patients with the central type, infiltration could be seen near the wall of the lesion, penetrating the wall of the cavity; the infiltration range in 23 patients exceeded 1/2 of the tube wall, and 80% of patients had mild enhancement. Therefore, CT can be used to determine the location of the tumor, its extension, the extent of invasion, and intrapulmonary metastases, which is important in guiding treatment plans. And it is believed that positron emission tomography-computed tomography plays a certain role in the delimitation of residual tumors and the target area of radiotherapy, and other imaging diagnostic manifestations and values need to be further evaluated [12]. In addition, Kim et al. [22] divided 104 PACC patients into surgery group, bronchial intervention group and other treatment groups and observed the prognosis, specifically 48 PACC patients accepted surgery, including 12 patients underwent surgery after bronchoscopic palliative treatment, 45 PACC patients received bronchoscopic intervention alone, and 11 patients received other treatments, and the results showed that there was no significant difference in overall survival of PACC patients between the surgical group and the bronchial intervention group in patients with stages III and IV. This study suggests that bronchoscopy can help determine treatment options and prolong survival by determining tumor shape, size, and intraluminal invasion, as well as biopsy to confirm diagnosis, while alleviating acute symptoms and providing patients who cannot tolerate surgery with the opportunity to operate.

## Pathological and immunohistochemical examinations

PACC is no capsule, medium hardness, and a median size of about 3–4 cm [14]. Lesions were also grayish white and the boundaries were unclear, and is mainly composed of ductal epithelial cells and mutated myoepithelial cells [23]. According to the tissue growth pattern of PACC, it can be divided into grade I tubular, grade II cribriform, and grade III solid tumors [17]. Cytological findings include tumor cells that are uniform in size and shape, with scant cytoplasm, and small and hyperchromatic nuclei. The most obvious feature is that tumor cells are arranged in sieve, glandular, cord-like, and solid nests and a dilated pseudocyst could be seen inside [2, 24]. The epidemiological characteristics and histopathological features of PACC are summarized in Table 1 [1–3, 9–11, 23–30]. As shown in Table 1, the cribriform and tubular type is relatively common, and the solid form is rare. The solid type is more malignant and more likely to invade surrounding tissues, leading to progression. In addition, some PACC patients have been reported to have mixed pathology, which will make proper diagnosis and grading more difficult.

**Table 1:** The reported clinical characteristics and histopathological features of PACC

Ref.	No. of cases	Age	sex		smoking	Location	stage		Histopathological Features			
		Median (range)	Male	Female		main bronchus	Stage I/II	Stage III/IV	tubular	Cribriform	Solid	Mixed
[1]	24	50.8(24 ~ 74)	7	17	6	24	NM	NM	NM	NM	NM	NM
[2]	4	NM	1	3	NM	NM	NM	NM	NM	NM	NM	NM
[3]	12	50.5(33 ~ 78)	7	5	6	8	NM	NM	NM	NM	NM	NM
[9]	34	46(22 ~ 73)	16	18	11	21	17	17	NM	NM	NM	NM
[10]	4	66(50 ~ 78)	3	1	2	3	3	1	NM	NM	NM	NM
[11]	21	49(24 ~ 69)	6	15	NM	15	NM	NM	5	8	0	8
[23]	40	56.6(21.6 ~ 73.4)	16	24	NM	NM	NM	NM	6	31	3	0
[24]	11	57(42 ~ 75)	6	5	9	NM	3	7	0	9	2	0
[25]	25	45(23 ~ 76)	11	14	4	NM	NM	NM	NM	NM	NM	NM
[26]	7	63(51 ~ 81)	3	4	3	7	NM	NM	3	4	0	0
[27]	12	47.5(26 ~ 64)	6	6	2	4	10	2	0	12	0	0
[28]	15	48(32 ~ 64)	5	10	13	13	11	4	NM	NM	NM	NM
[29]	49	NM	24	25	NM	NM	NM	NM	9	36	4	0
[30]	1	30	0	1	0	1	NM	NM	0	1	0	0

Abbreviations: NM, not mention

Immunohistochemically, myoepithelial markers (p63, S-100, and calponin) and vimentin in basal cells are usually positive, while luminal cells are positive for CK7, SMA, CK5/6, and CD117. The IHC markers of PACC were retrospectively analyzed, and the results are shown in Table 2 [3, 9–11, 17, 29–32]. As shown in Table 2, the positivity percentages of CK7, CD117, P63, SMA, CK5/6, S-100, TTF-1, CK20, CgA, CD56, and napsin A are 100% (15/15), 94.4% (67/71), 92.0% (46/50), 90.0% (27/30), 83.3% (5/6), 80.0% (8/10), 7.8% (4/51), 0 (0/4), 0 (0/5), 0 (0/10) and 0 (0/2), respectively. In addition, High expression levels of CK7, CD117, P63, SMA, CK5/6, and S-100 are helpful for the diagnosis of PACC. Ki-67, a reliable marker of tumor cell proliferative activity, often shows different degrees of positivity (2-35%) in PACC, and the expression level in solid type is generally higher than in other types [11]. TTF-1 and napsin A are highly sensitive and specific markers expressed in primary lung adenocarcinoma [3]. Therefore, low expression rates of napsin A and TTF-1 in PACC indicate that these lesions derive from other lung phenotypes. It is worth noting that CD117 positivity has a special role in the diagnosis of PACC, especially when CD117 is combined with the MYB protein due to PACC-specific MYB chromosomal translocation [2]. In addition, it has been reported that CD117 positivity in the myoepithelial cells of the lesion may indicate low differentiation of myoepithelial cells, which is associated with a poor prognosis. In general, CD117 can help differentiate between PACC and common lung cancer subtypes such as lung adenocarcinoma, but it cannot be used alone for the differential diagnosis of salivary gland tumors, because other salivary gland tumors can also express CD117 to varying degrees [33].

**Table 2:** The frequency of immunohistochemical results of PACC

Ref.	IHC result(No.of patients)										
	CK7	CD117	P63	SMA	CK5/6	S-100	TTF-1	CK20	CgA	CD56	Napsin A
[3]	NM	9/12	12/12	NM	NM	NM	4/12	NM	NM	NM	NM
[9]	11/11	NM	11/12	6/9	1/1	7/8	0/14	0/4	0/4	0/7	NM
[10]	1/1	2/2	0/2	NM	1/2	NM	0/2	NM	NM	0/1	NM
[11]	NM	20/20	20/20	20/20	NM	NM	0/20	NM	NM	NM	NM
[17]	1/1	1/1	0/1	NM	1/1	0/1	0/1	NM	NM	0/1	0/1
[29]	NM	33/34	NM	NM	NM	NM	NM	NM	NM	NM	NM
[30]	1/1	1/1	1/1	1/1	1/1	1/1	0/1	NM	NM	NM	NM
[31]	NM	NM	1/1	NM	1/1	NM	0/1	NM	0/1	0/1	0/1
[32]	1/1	1/1	1/1	NM	NM	NM	NM	NM	NM	NM	NM
Total	15/15	67/71	46/50	27/30	5/6	8/10	4/51	0/4	0/5	0/10	0/2
Rate	100%	94.4%	92.0%	90.0%	83.3%	80%	7.8%	0	0	0	0

IHC results: Patients who express the biomarker/all patients who tested IHC in the reference (positive frequency). NM: not mention

## Molecular characteristics

The most obvious genomic characteristic of adenoid cystic carcinoma (ACC) is t(6;9)(q22-23;p23-24) translocation, a translocation of the v-myb avian myeloid virus oncogene homolog (MYB) gene, resulting in the fusion of the gene encoding transcription factor MYB with the nuclear factor IB (NFIB) transcription factor and leading to overexpressing MYB gene and its downstream target genes, and involved in cell cycle regulation, cell growth, apoptosis and cell adhesion [34–38]. The mutational profile of PACC differs significantly from other ACCs, which has a signature MYB gene translocation, occurring in approximately 80% of ACCs. However, the incidence of MYB gene translocation in PACC is only about 40% [23, 35, 39]. In patients without MYB-NFIB fusion, there were an alternative genetic mechanism or MYB overexpression. Pei et al. [26] assessed 7 PACC cases and found MYB-NFIB gene fusion in 3 patients; of the remaining 4 cases, 3 had MYBL1-NFIB gene fusion and 1 had a rare fusion, MYBL1-RAD51B. The structure of the MYB-NFIB fusion gene is very similar to that of the MYBL1-NFIB fusion gene. The proteins encoded by the MYB and MYBL1 genes have nearly identical DNA binding domains and similar overall structures, suggesting that the associated MYB proteins are interchangeable oncogenic drivers in adenoid cystic carcinoma. In addition, genetic mutations of the Notch pathway are present in about 25% of ACC and most commonly mutations in NOTCH1 [39]. Less commonly, NOTCH2-4 mutations are found, as well as genes encoding key downstream proteins in the signaling pathway, such as LPAR3 and ALPI genes [25]. In addition, Ferrarotto et al. [40] found that upregulation of Notch signaling suppress myoepithelial differentiation in ACC, a characteristic of the solid subtype, so NOTCH1 mutation is likely to promote the formation of solid subtype and associated with increased rates of liver and bone metastasis and poor prognosis in PACC.

Bell et al. [41] detected gene mutations in 16 patients with ACC of lacrimal glands in 2016, and found that the KRAS gene mutation rate (31.3%) was high, which is different from the mutation characteristics of PACC. In PACC patients, the rates of EGFR, ALK and KRAS mutations are low. Huo et al. [1] used next-generation sequencing, sanger sequencing, and quantitative polymerase chain reaction to analyze mutations in 9 PACC patients, and the analysis revealed no mutations in the EGFR, KRAS, BRAF, ALK, PIK3CA, PDGFRA and DDR2 genes. Similarly, Li et al. [6] performed gene sequencing on surgical samples from 8 PACC patients and showed that the most mutations were KAT6A (4/8), KMT2D (3/8), and TET2 (3/8), and there were no common lung cancer mutations such as EGFR, ALK, and KRAS. In addition, previous studies have also found that the median tumor mutation burden of PACC is lower than that of other solid tumors [25, 42]. These studies suggest that the molecular characteristics of PACC patients may differ from those of ACC originating from other sites. However, the incidence rates of EGFR and other mutations may be different among different races and distinct pathological types [43], and its demonstration requires more genetic analysis studies in PACC patients.

## Diagnosis and differential diagnosis

The time of diagnosis from symptom onset ranges from 1 to 84 months (median, 7 months), and some early cases are misdiagnosed as other respiratory diseases and treated incorrectly, because of atypical symptoms in PACC patients [11, 44]. Thus, in order to avoid misdiagnosis due to atypical clinical symptoms, early CT examination and bronchial examination are particularly important. Other examinations, including fine needle aspiration, bronchial aspiration or scrub, bronchoalveolar lavage and even sputum cytology, have proven to be effective diagnostic tools for lung cancer, particularly in tumors like PACC, which often invade the central bronchi and present with intra bronchial growth [2].

Differentiation between PACC and primary lung adenocarcinoma is relatively simple. PACC is more likely to occur in the trachea or main bronchus, whereas conventional lung adenocarcinoma is more likely to occur around the lung. Moreover, IHC showed that PACC can express specific markers, including CD117, SMA, P63 and S-100, which is not the case for primary lung adenocarcinoma that is usually TTF-1 negative. In addition, poorly differentiated squamous cell carcinoma is morphologically easy to mix with solid PACC, and its IHC showed that P63 and CK5/6 are also positive, but CD117 is negative. Therefore, PACC and poorly differentiated squamous cell carcinoma can be distinguished by the expression of CD117. In conclusion, PACC can be distinguished from primary lung cancer through morphology and IHC [2, 9, 11].

Studies have shown that PACC is the second most common pathological type of pulmonary salivary gland tumors [30]. The differential diagnosis of PACC and other salivary gland tumors such as metastatic adenoid cystic carcinoma of salivary glands, basal cell adenomas, pleomorphic adenomas, and pulmonary mucoepidermoid carcinoma are very important for the diagnosis and treatment of PACC, which seriously affects clinical stage, treatment strategy and prognosis in PACC. First of all, metastatic adenoid cystic carcinoma of salivary glands, mainly of the parotid gland, is not difficult to identify based on its clinical history [19]. Then, basal cell adenoma and pleomorphic adenoma are benign tumors of bronchial salivary glands. Their pathological morphology can appear tubular or cribriform structure similar to PACC, and both can express epithelial and myoepithelial markers, but its border is clear and does not invade surrounding tissues, which is different from PACC [24, 45]. For the identification of pulmonary mucoepidermoid carcinoma and PACC, imaging analysis showed that PACC occurs in the trachea or main bronchus, while pulmonary mucoepidermoid carcinoma mostly occurs in segmental and lobular bronchi [46]. Pathologically, tumor cells in PACC are round or ovoid, like basal cells, and aggregated in granular form under a microscope. The mucus is granular and surrounded by one or more layers of tumor cells, whereas pulmonary mucoepidermoid carcinoma does not have this structure. In addition, the positive expressions of CD117, SMA, and S-100 are helpful for the differential diagnosis of PACC and pulmonary mucoepidermoid carcinoma. Furthermore, the MYB gene translocations are specific mutations in PACC, which is a key point to distinguish PACC from other salivary gland lung cancers [34, 39]. According to the above results, distinguishing PACC from primary lung adenocarcinoma is relatively simple. However, the differentiation of PACC from other salivary gland tumors is primarily based on histopathology and its specific gene fusion mutations.

## Treatment

**Fig. 1:**
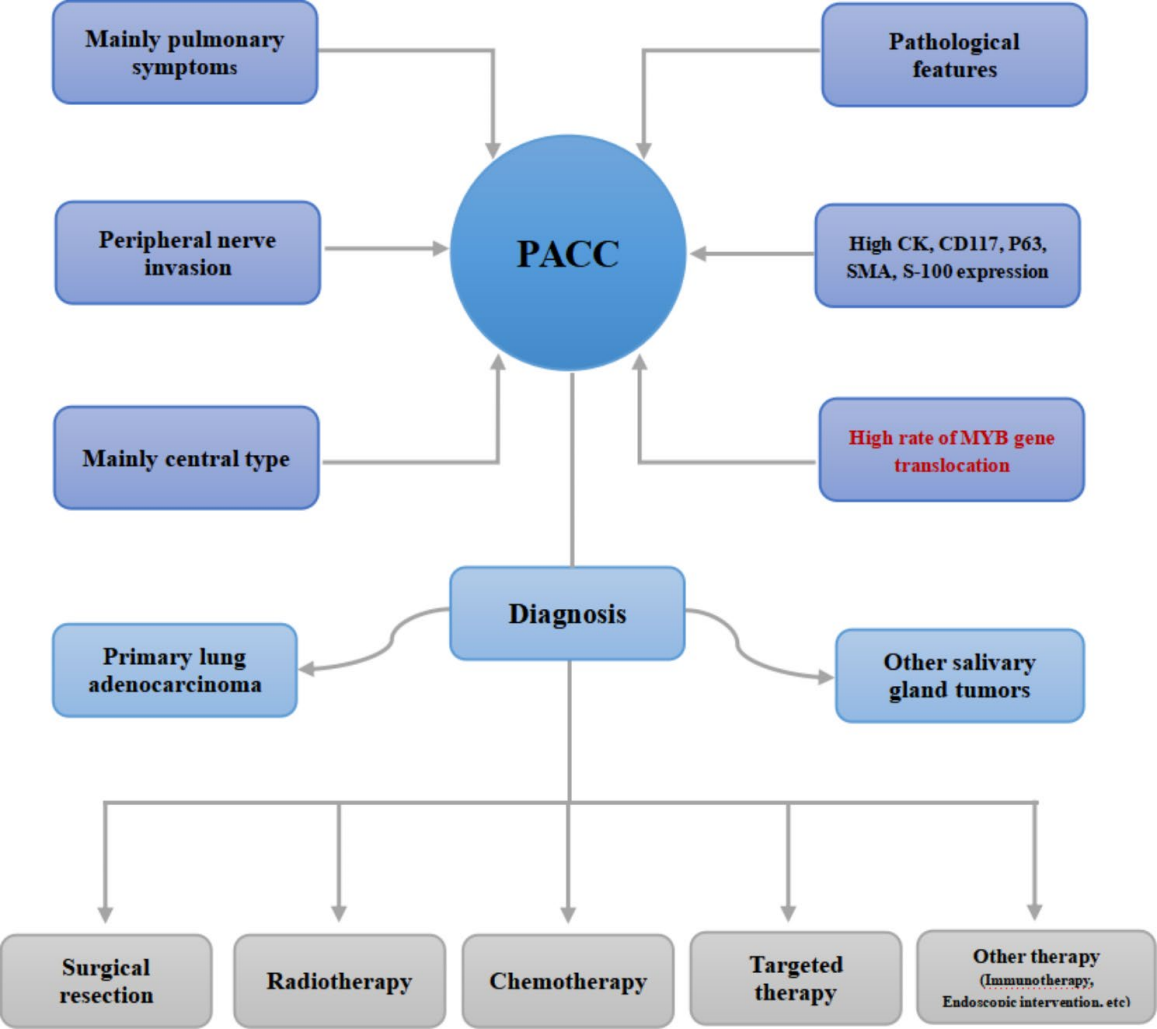
The diagnosis and treatment of PACC

The diagnosis and treatment process of PACC is shown in Fig. 1. Early-stage PACC patients can achieve satisfactory survival by receiving multimodality therapy. Surgical resection is the main therapy for PACC, and depending on the location and extent of the tumor, the types of surgery may include resection of the trachea, lobectomy, pneumonectomy and segmental resection [17, 31]. Local recurrence rates are relatively high in PACC patients with postoperative positive-margins, while endoscopic interventions are an option for palliative treatment in patients who are difficult or unwilling to undergo reoperation after recurrence. For example, Huang et al. [30] reported a case of inoperable patient whose lesions shrank, and the symptoms relieved after endoscopic interventional therapy. In addition, radiotherapy is usually recommended for patients with unresectable PACC, positive surgical margin indicated by surgical pathology, and postoperative in-situ recurrence that cannot be re-operated, with encouraging effectiveness [47]. However, in PACC cases who can be completely resected and show negative margins, it is still controversial whether the patient needs postoperative radiotherapy. Although progression-free survival of PACC patients accepted chemotherapy alone is only 4–6 months [10, 11, 40], chemotherapy remains one of the main treatment strategies for advanced PACC patients, and the platinum-containing dual-drug regimen recommended by the NSCLC guidelines is the main chemotherapy regimen for PACC, including carboplatin, cisplatin combined with pemetrexed, paclitaxel and gemcitabine [14]. Currently, other effective treatments are urgently needed.

In terms of targeted therapy, MYB and its downstream oncogenic effects are therapeutic targets for this disease. Therapies targeting the MYB activation pathway for PACC are being explored. For example, Jiang et al. [48] generated and studied new murine and human MYB-activated tumor samples and detected growth inhibition with MYB peptidomimetics. Yusenko et al. [49] found that monensin inhibits ACC cells expressing MYB-NFIB fusion oncoprotein and Andersson et al. [34] found that ATR, a target downstream of MYB, can use ATR kinase inhibitor to induce apoptosis in MYB positive ACC cells. These studies provide tools to define treatment strategies for patients with advanced MYB-activated PACC. In addition, it was reported that targeting c-kit (a downstream effector of MYB) also can provide an alternative approach for PACC treatment [26]. Bhattacharyya et al. [50] reported that a c-kit positive PACC patient treated with imatinib (a c-kit inhibitor), the best response achieved partial response and PFS achieved 12 months, suggesting that targeting MYB downstream genes is also effective. However, Dillon et al. [51] reported that only 2 of 42 head and neck ACC patients treated with imatinib had objective tumor response, and the reason for the significant difference in the results of above trials may be that inhibiting MYB and its downstream genes is more effective in primary pulmonary ACC than in other sites.

For a small number of PACC patients with EGFR mutation, tyrosine kinase inhibitor (TKIs) therapy is effective and the reported targeted therapy cases are shown in Table 3 [10, 31, 43, 50, 52, 53]. Song et al. [53] reported a PACC patient receiving first-line chemotherapy with regimen of pemetrexed and cisplatin who discontinued chemotherapy after only 2 months, because disease progression of new liver lesions, and then a mutation in EGFR exon 19 was determined using genetic testing, after which he received icotinib and PFS achieved 19 months without significant adverse events. This case may suggest that carrying driver gene mutations may be one of the reasons for the poor response to chemotherapy in PACC patients. In a multicenter phase 2 clinical trial of ACC patients [54], a total of 20 patients participated in the trial and accepted VEGFR-TKIs; although no patients achieved partial response, the disease-control rate reached 75% (15/20). Similarly, He et al. [10] reported that an advance PACC patient treated with chemotherapy combined VEGFR-TKIs, and stable disease achieved 12 months. These results show that targeted therapy for PACC patients with sensitive mutations has obvious effects, indicating that the development and use of targeted drugs targeting the MYB or its related genes may also achieve significant efficacy in MYB-activated PACC.

**Table 3:** The reported targeted therapy cases of PACC

Ref.	Sex; Age; Smoking	Gene mutation	Targeted therapy	Response	Outcomes	PFS (mo)	OS (mo)
[10]	F; 50; No	No	Bevacizumab	SD	Alive	About 12	About 48
[31]	F; 37; No	ERBB (+)	Pyrotinib	SD	Alive	6	About 168
[43]	F; 80; NM	EGFR exon 18 (+)	Gefitinib	PR	Died	6	19
[50]	M; 42; Yes	c-kit (+)	Imatinib	PR	Alive	12	NM
[52]	F; 60; No	EGFR exon 21 (+)	Erlotinib	SD	Died	8	33
[53]	M; 29; No	EGFR exon 19 (+)	Icotinib	SD	Alive	19	179

M male, F female, SD stable disease, PD progressive disease, PFS progression-free survival, OS overall survival, NM not mention

In 2015, Rizvi et al. [55] found an obvious correlation between tumor mutation burden and the sensitivity of lung cancer cases to PD-1 blockade. However, the tumor mutation burden of PACC is lower than those of other solid tumors, and PD-L1 is often not expressed or only low expressed in PACC patients [25], which indicate that immune checkpoint inhibitors alone might not be a useful treatment for PACC. Actually, Rodriguez et al. [56] reported that the combined treatment of pembrolizumab and vorinostat in 25 patients with salivary gland cancer, including 12 ACC patients, was disappointing, with low response rates. In recent years, it was shown that PD-L2 expression is high in ACC, suggesting that PD-1/PD-L2 may be a new pathway for tumor cell immune evasion, especially in ACC, which deserves further study [57]. In addition, in a trial using wilm’s tumor 1 peptide vaccine to treat ACC patients with pulmonary metastasis, tumor growth was significantly inhibited within one year of treatment; after stopping the treatment the tumors grew rapidly, and new metastases rapidly appeared [58]. This indicates that cancer vaccines may be effective in PACC. Similarly, because MYB gene translocations are oncogenic drivers in PACC, the MYB protein may be an ideal target for developing vaccines against PACC.

## Prognosis

Due to the rarity of PACC cases, the sample sizes of relevant reports are small, and the survival of patients varies greatly after surgery [14, 23, 59]. Factors that may influence the prognosis of PACC include tumor stage, location, positive surgical margin and treatment modality. Zhao et al. [14] analyzed the prognosis of 35 patients who received surgery, and the results showed that the 5 years survival of patients with negative surgical margins was significantly longer than for those with positive surgical margins (R0 vs. R1: 94.4% vs. 66.0%, P = 0.014). In addition, most of patients (9/15) with positive surgical margins received adjuvant radiotherapy, which might have contributed to prolonged survival. Currently, multiple studies have focused on the effects of different treatments on the prognosis of PACC. Hogerle et al. [59] reported the prognosis of 38 patients who were divided into three groups, surgery alone, radiotherapy alone, and surgery plus adjuvant radiotherapy, and the results showed that 80%, 67%, and 65% of patients in the surgery, radiotherapy and surgery plus adjuvant radiotherapy groups, respectively, had no distant progression within 5 years. This suggests that early surgery is still the best therapy option. In addition, postoperative PACC patients with high-risk factors, such as positive resection margins, lymph node metastases, and pathologic results of poorly differentiated were more likely to accepted surgery plus radiotherapy, and they achieved longer survival than patients who accepted surgery alone [60].

Xu et al. [61] collected 50 ACC tissues and 41 normal glandular tissues, respectively tested MYB mRNA expression, compared with normal glandular tissue, MYB expression in ACC tissues increased significantly, and MYB was negatively correlated with CDH1 (the gene that encodes cadherin‑1) and positively correlated with VIM (the gene that encodes vimentin), suggesting that MYB induce epithelial-mesenchymal transformation and was related to ACC metastasis, in addition, the researchers injected MYB overexpression cells and normal cells into 7 and 6 mice through veins, respectively, and sacrificed mice 8 weeks later to obtain their lung tissue and the results also prove that MYB promotes lung metastasis of ACC. Similarly, it has been reported that solid histological type and MYB-NFIB balanced translocation mutation are associated with poor prognosis in PACC patients [15, 62].

## Conclusion

PACC is an exceptionally rare salivary gland-type malignant neoplasm. Accurate and early diagnosis is crucial for the treatment and prognosis of PACC patients. CT and bronchoscopy examination is important for diagnosis and treatment plan design in PACC. High expression levels of CK7, CD117, P63, SMA, CK5/6, and S-100 are helpful in the diagnosis of PACC. Because of low incidence and limited case information, a standard treatment model is lacking. Surgery, radiotherapy and chemotherapy remain the main treatment options for PACC. The high rate of MYB gene rearrangement and its downstream genes carcinogenic effects in PACC still makes targeted therapy a hot topic for PACC therapy, which is worthy of further study. Advanced PACC patients are less likely to benefit from immune checkpoint inhibitors alone due to low tumor mutation burden and negative or low PD-L1 expression, but immune checkpoint inhibitors in combination with chemoradiotherapy and targeted therapy may be beneficial. In addition, the prognostic parameters related to predominant histological pattern, tumor staging, surgical margin status and MYB translocation mutations and overexpression, etc. At present, little information is currently available on PACC, so further research is needed to further our understanding of PACC.

## Data Availability

The datasets used and analysed during the current study are available from the corresponding author on reasonable request.

## Abbreviations

PACC: Pulmonary adenoid cystic carcinoma
ACC: Adenoid cystic carcinoma
IHC: Immunohistochemical
MYB: V-myb avian myeloblastosis virus oncogene homolog
NFIB: Nuclear factor IB
CT: Computed tomography
TKIs: Tyrosine kinase inhibitors

## References

[1] Huo Z, Wu H, Li S, Liang Z (2015). Molecular genetic studies on EGFR, KRAS, BRAF, ALK, PIK3CA, PDGFRA, and DDR2 in primary pulmonary adenoid cystic carcinoma. Diagn Pathol.

[2] Saglietti C, Volante M, La Rosa S, Letovanec I, Pusztaszeri M, Gatti G, Bongiovanni M (2017). Cytology of primary salivary gland-type tumors of the Lower Respiratory Tract: report of 15 cases and review of the literature. Front Med (Lausanne).

[3] Qing S, Zhou K, Liu X, Li X, Deng F, Ma Y (2015). Primary pulmonary adenoid cystic carcinoma: clinicopathological analyses of 12 cases. Int J Clin Exp Pathol.

[4] West RB, Kong C, Clarke N, Gilks T, Lipsick JS, Cao H, Kwok S, Montgomery KD, Varma S, Le QT (2011). MYB expression and translocation in adenoid cystic carcinomas and other salivary gland tumors with clinicopathologic correlation. Am J Surg Pathol.

[5] Krifa M, Bdioui A, Lajmi Z, Missaoui N, Hmissa S, Mokni M (2021). Primary adenoid cystic carcinoma of the lung: a case report and literature review. Heliyon.

[6] Li M, Zhao BR, Liu SQ, An J (2018). Mutational landscape and clonal diversity of pulmonary adenoid cystic carcinoma. Cancer Biol Ther.

[7] Li N, Xu L, Zhao H, El-Naggar AK (2012). A comparison of the demographics, clinical features, and survival of patients with adenoid cystic carcinoma of major and minor salivary glands versus less common sites within the Surveillance, Epidemiology, and end results registry. Cancer.

[8] Elnayal A, Moran CA, Fox PS, Mawlawi O, Swisher SG, Marom EM (2013). Primary salivary gland-type lung cancer: imaging and clinical predictors of outcome. AJR Am J Roentgenol.

[9] Hu MM, Hu Y, He JB, Li BL (2015). Primary adenoid cystic carcinoma of the lung: clinicopathological features, treatment and results. Oncol Lett.

[10] He X, Chen J (2017). [Four cases report on primary lung adenoid cystic Carcinoma]. Zhongguo Fei Ai Za Zhi.

[11] Huo Z, Meng Y, Wu H, Shen J, Bi Y, Luo Y, Cao J, Liang Z (2014). Adenoid cystic carcinoma of the tracheobronchial tree: clinicopathologic and immunohistochemical studies of 21 cases. Int J Clin Exp Pathol.

[12] Ran J, Qu G, Chen X, Zhao D (2021). Clinical features, treatment and outcomes in patients with tracheal adenoid cystic carcinoma: a systematic literature review. Radiat Oncol.

[13] Molina JR, Aubry MC, Lewis JE, Wampfler JA, Williams BA, Midthun DE, Yang P, Cassivi SD (2007). Primary salivary gland-type lung cancer: spectrum of clinical presentation, histopathologic and prognostic factors. Cancer.

[14] Zhao Y, He G, Zhai Y, Zhou Z, Bi N, Mao Y, Zhang Y, Xiao Z, Gao S, Lv J (2022). Adenoid cystic carcinoma of Lobar Bronchial Origin: 20-Year experience at a single Institution. Ann Surg Oncol.

[15] Ju J, Li Y, Chai J, Ma C, Ni Q, Shen Z, Wei J, Sun M (2016). The role of perineural invasion on head and neck adenoid cystic carcinoma prognosis: a systematic review and meta-analysis. Oral Surg Oral Med Oral Pathol Oral Radiol.

[16] Aldrees T, Alanazi A, Fatani HA, Samman A, Aldhahri SF (2016). Adenoid cystic carcinoma of the upper airway mimicking a thyroid tumor: a case report. Mol Clin Oncol.

[17] Gao F, Zang L, He J, Xu W. A Case of Solid Variant of Adenoid Cystic Carcinoma from Trachea: A Case Report and Review of Literature. OncoTargets and Therapy 2021 Volume 14:1997–2002. doi:10.2147/ott.S296400. 10.2147/OTT.S296400 PMC7987321 33776449

[18] Resio BJ, Chiu AS, Hoag J, Dhanasopon AP, Blasberg JD, Boffa DJ (2018). Primary salivary type lung cancers in the National Cancer Database. Ann Thorac Surg.

[19] Nightingale J, Lum B, Ladwa R, Simpson F, Panizza B (2021). Adenoid cystic carcinoma: a review of clinical features, treatment targets and advances in improving the immune response to monoclonal antibody therapy. Biochim Biophys Acta Rev Cancer.

[20] Junejo NN, Almusalam L, Alothman KI, Al Hussain TO (2019). An unusual case report of pulmonary adenoid cystic carcinoma metastasis to the kidney. Case report and literature review. Urol Case Rep.

[21] Li X, Yi W, Zeng Q (2018). CT features and differential diagnosis of primary pulmonary mucoepidermoid carcinoma and pulmonary adenoid cystic carcinoma. J Thorac Dis.

[22] Kim BG, Lee K, Um SW, Han J, Cho JH, Kim J, Kim H, Jeong BH (2020). Clinical outcomes and the role of bronchoscopic intervention in patients with primary pulmonary salivary gland-type tumors. Lung Cancer.

[23] Roden AC, Greipp PT, Knutson DL, Kloft-Nelson SM, Jenkins SM, Marks RS, Aubry MC, Garcia JJ (2015). Histopathologic and cytogenetic features of Pulmonary Adenoid cystic carcinoma. J Thorac Oncol.

[24] Kim S, Chu J, Kim H, Han J (2015). Comprehensive Cytomorphologic Analysis of Pulmonary Adenoid cystic carcinoma: comparison to small cell carcinoma and non-pulmonary adenoid cystic carcinoma. J Pathol Transl Med.

[25] Wang F, Xie X, Song M, Ji L, Liu M, Li P, Guan Y, Lin X, Qin Y, Xie Z (2020). Tumor immune microenvironment and mutational analysis of tracheal adenoid cystic carcinoma. Ann Transl Med.

[26] Pei J, Flieder DB, Patchefsky A, Talarchek JN, Cooper HS, Testa JR, Wei S (2019). Detecting MYB and MYBL1 fusion genes in tracheobronchial adenoid cystic carcinoma by targeted RNA-sequencing. Mod Pathol.

[27] Zhu F, Liu Z, Hou Y, He D, Ge X, Bai C, Jiang L, Li S (2013). Primary salivary gland-type lung cancer: clinicopathological analysis of 88 cases from China. J Thorac Oncol.

[28] Han X, Zhang J, Fan J, Cao Y, Gu J, Shi H. Radiological and Clinical Features and Outcomes of Patients with Primary Pulmonary Salivary Gland-Type Tumors. Can Respir J 2019,2019:1475024. doi:10.1155/2019/1475024. 10.1155/2019/1475024 PMC6466881 31065298

[29] Aubry MC, Heinrich MC, Molina J, Lewis JE, Yang P, Cassivi SD, Corless CL (2007). Primary adenoid cystic carcinoma of the lung: absence of KIT mutations. Cancer.

[30] Huang HC, Zhao L, Cao XH, Meng G, Wang YJ, Wu M (2021). Primary salivary gland tumors of the lung: two cases date report and literature review. Respir Med Case Rep.

[31] Tang Z, Lin F, Xiao J, Du X, Zhang J, Li S, Tang G, Chen C, Li J (2021). Case Report: efficacy of Pyrotinib in ERBB2 amplification Pulmonary Adenoid cystic carcinoma. Front Oncol.

[32] Philippou P, Michalakis A, Miliatou M, Poullou C, Constantinou P (2021). Solitary renal metastasis arising from a pulmonary adenoid cystic carcinoma: a Case Report and Review of the literature. Case Rep Urol.

[33] Roden AC (2021). Recent updates in salivary gland tumors of the lung. Semin Diagn Pathol.

[34] Andersson MK, Mangiapane G, Nevado PT, Tsakaneli A, Carlsson T, Corda G, Nieddu V, Abrahamian C, Chayka O, Rai L (2020). ATR is a MYB regulated gene and potential therapeutic target in adenoid cystic carcinoma. Oncogenesis.

[35] Xie M, Wu X, Zhang J, He C, Wei S, Huang J, Fu X, Gu Y (2018). The Prognostic significance of Notch1 and fatty acid binding protein 7 (FABP7) expression in Resected Tracheobronchial Adenoid cystic carcinoma: a Multicenter Retrospective Study. Cancer Res Treat.

[36] Gao R, Cao C, Zhang M, Lopez MC, Yan Y, Chen Z, Mitani Y, Zhang L, Zajac-Kaye M, Liu B (2014). A unifying gene signature for adenoid cystic cancer identifies parallel MYB-dependent and MYB-independent therapeutic targets. Oncotarget.

[37] Chae YK, Chung SY, Davis AA, Carneiro BA, Chandra S, Kaplan J, Kalyan A, Giles FJ (2015). Adenoid cystic carcinoma: current therapy and potential therapeutic advances based on genomic profiling. Oncotarget.

[38] Drier Y, Cotton MJ, Williamson KE, Gillespie SM, Ryan RJ, Kluk MJ, Carey CD, Rodig SJ, Sholl LM, Afrogheh AH (2016). An oncogenic MYB feedback loop drives alternate cell fates in adenoid cystic carcinoma. Nat Genet.

[39] Thierauf J, Ramamurthy N, Jo VY, Robinson H, Frazier RP, Gonzalez J, Pacula M, Dominguez Meneses EM, Nose V, Nardi V (2019). Clinically Integrated Molecular Diagnostics in Adenoid cystic carcinoma. Oncologist.

[40] Ferrarotto R, Mitani Y, Diao L, Guijarro I, Wang J, Zweidler-McKay P, Bell D, William WN, Glisson BS, Wick MJ (2017). Activating NOTCH1 mutations define a distinct subgroup of patients with adenoid cystic Carcinoma who have poor prognosis, propensity to bone and liver metastasis, and potential responsiveness to Notch1 inhibitors. J Clin Oncol.

[41] Bell D, Sniegowski MC, Wani K, Prieto V, Esmaeli B (2016). Mutational landscape of lacrimal gland carcinomas and implications for treatment. Head Neck.

[42] Ho AS, Ochoa A, Jayakumaran G, Zehir A, Valero Mayor C, Tepe J, Makarov V, Dalin MG, He J, Bailey M (2019). Genetic hallmarks of recurrent/metastatic adenoid cystic carcinoma. J Clin Invest.

[43] Fujita M, Matsumoto T, Hirano R, Uchino J, Hirota T, Yamaguchi E (2016). Adenoid cystic carcinoma of the lung with an EGFR mutation. Intern Med.

[44] Saad AB, Kadoussi R, Njima M, Mhamed SC, Fahem N, Abdeljelil NB, Joobeur S, Rouatbi N (2019). Primary adenoid cystic carcinoma of the tracheobronchial tree: report of two cases. Pan Afr Med J.

[45] Alzumaili B, Xu B, Saliba M, Abuhashem A, Ganly I, Ghossein R, Katabi N (2022). Clinicopathologic characteristics and prognostic factors of primary and recurrent pleomorphic adenoma: a single Institution Retrospective Study of 705 cases. Am J Surg Pathol.

[46] Kumar V, Soni P, Garg M, Goyal A, Meghal T, Kamholz S, Chandra AB (2018). A comparative study of primary adenoid cystic and mucoepidermoid carcinoma of lung. Front Oncol.

[47] Hashimoto S, Sumida Y, Tobinaga S, Wada H, Wakata K, Nonaka T, Kunizaki M, Hidaka S, Kinoshita N, Sawai T (2018). Liver resection for metastases of tracheal adenoid cystic carcinoma: report of two cases. Int J Surg Case Rep.

[48] Jiang Y, Gao R, Cao C, Forbes L, Li J, Freeberg S (2019). MYB-activated models for testing therapeutic agents in adenoid cystic carcinoma. Oral Oncol.

[49] Yusenko MV, Trentmann A, Andersson MK, Ghani LA, Jakobs A, Arteaga Paz MF, Mikesch JH, von Peter J, Stenman G, Klempnauer KH (2020). Monensin, a novel potent MYB inhibitor, suppresses proliferation of acute myeloid leukemia and adenoid cystic carcinoma cells. Cancer Lett.

[50] Bhattacharyya T, Bahl A, Kapoor R, Bal A, Das A, Sharma SC (2013). Primary adenoid cystic carcinoma of lung: a case report and review of the literature. J Cancer Res Ther.

[51] Dillon PM, Chakraborty S, Moskaluk CA, Joshi PJ, Thomas CY (2016). Adenoid cystic carcinoma: a review of recent advances, molecular targets, and clinical trials. Head Neck.

[52] Mendes MA, Barroso A, Campainha S (2018). EGFR-Variant adenoid cystic carcinoma of the lung. J Thorac Oncol.

[53] Song Z, Wu W, Zhang Y (2014). Effective treatment with icotinib in primary adenoid cystic carcinoma of the lung with liver metastasis. J Thorac Oncol.

[54] Kim Y, Lee SJ, Lee JY, Lee SH, Sun JM, Park K, An HJ, Cho JY, Kang EJ, Lee HY (2017). Clinical trial of nintedanib in patients with recurrent or metastatic salivary gland cancer of the head and neck: a multicenter phase 2 study (Korean Cancer Study Group HN14-01). Cancer.

[55] Rizvi NA, Hellmann MD, Snyder A, Kvistborg P, Makarov V (2015). Cancer immunology. Mutational landscape determines sensitivity to PD-1 blockade in non-small cell lung cancer. Science.

[56] Rodriguez CP, Wu QV, Voutsinas J, Fromm JR, Jiang X, Pillarisetty VG, Lee SM, Santana-Davila R, Goulart B, Baik CS (2020). A phase II trial of Pembrolizumab and Vorinostat in Recurrent Metastatic Head and Neck squamous cell carcinomas and salivary gland Cancer. Clin Cancer Res.

[57] Mosconi C, de Arruda JAA, de Farias ACR, Oliveira GAQ, de Paula HM, Fonseca FP, Mesquita RA, Silva TA, Mendonca EF, Batista AC (2019). Immune microenvironment and evasion mechanisms in adenoid cystic carcinomas of salivary glands. Oral Oncol.

[58] Sasabe E, Hamada F, Iiyama T, Udaka K (2011). Wilm’s tumor gene WT1 peptide immunotherapy for pulmonary metastasis from adenoid cystic carcinoma of the salivary gland. Oral Oncol.

[59] Hogerle BA, Lasitschka F, Muley T, Bougatf N, Herfarth K, Adeberg S, Eichhorn M, Debus J, Winter H, Rieken S (2019). Primary adenoid cystic carcinoma of the trachea: clinical outcome of 38 patients after interdisciplinary treatment in a single institution. Radiat Oncol.

[60] Yang Y, Ran J, Wang Y, Zhou Z, Chen D, Feng Q, Liang J, Xiao Z, Hui Z, Lv J (2021). Intensity modulated radiation therapy may improve survival for tracheal-bronchial adenoid cystic carcinoma: a retrospective study of 133 cases. Lung Cancer.

[61] Xu LH, Zhao F, Yang WW, Chen CW, Du ZH, Fu M, Ge XY, Li SL (2019). MYB promotes the growth and metastasis of salivary adenoid cystic carcinoma. Int J Oncol.

[62] Han J, Zhang C, Gu T, Yang X, Hu L, Tian Z, Li J, Zhang C (2019). Analysis of clinicopathological characteristics, MYB rearrangement and prognostic factors in salivary adenoid cystic carcinoma. Oncol Lett.

